# Transcutaneous Electrical Neuromodulation of the Cervical Spinal Cord Depends Both on the Stimulation Intensity and the Degree of Voluntary Activity for Training. A Pilot Study

**DOI:** 10.3390/jcm10153278

**Published:** 2021-07-25

**Authors:** Hatice Kumru, María Rodríguez-Cañón, Victor R. Edgerton, Loreto García, África Flores, Ignasi Soriano, Eloy Opisso, Yury Gerasimenko, Xavier Navarro, Guillermo García-Alías, Joan Vidal

**Affiliations:** 1 Fundación Institut Guttmann, Institut Universitari de Neurorehabilitació Adscrit a la UAB, 08916 Badalona, Spain; rodriguezcanonmaria@gmail.com (M.R.-C.); vre@ucla.edu (V.R.E.); loretogarcia@guttmann.com (L.G.); isoriano@guttmann.com (I.S.); eopisso@guttmann.com (E.O.); xavier.navarro@uab.cat (X.N.); guillermo.garcia@uab.cat (G.G.-A.); jvidal@guttmann.com (J.V.); 2 Universitat Autònoma de Barcelona, 08193 Barcelona, Spain; 3 Fundació Institut d’Investigació en Ciències de la Salut Germans Trias i Pujol, 08916 Badalona, Spain; 4 Departament de Biologia Cel·lular, Fisiologia i Immunologia & Insititute of Neuroscience, Universitat Autònoma de Barcelona, 08193 Bellaterra, Spain; africa.flores@uab.cat; 5 Department of Neurobiology, University of California Los Angeles, Los Angeles, CA 90095, USA; 6 Pavlov Institute of Physiology, 199034 St. Petersburg, Russia; yury.gerasimenko@louisville.edu; 7 Department of Physiology and Biophysics, University of Louisville, Louisville, KY 40292, USA; 8 Kentucky Spinal Cord Injury Research Center, University of Louisville, Louisville, KY 40292, USA

**Keywords:** transcutaneous spinal cord stimulation, intensity effect, muscle strength effect, hand training, neuromodulation, cervical spinal cord

## Abstract

Electrical enabling motor control (eEmc) through transcutaneous spinal cord stimulation offers promise in improving hand function. However, it is still unknown which stimulus intensity or which muscle force level could be better for this improvement. Nine healthy individuals received the following interventions: (i) eEmc intensities at 80%, 90% and 110% of abductor pollicis brevis motor threshold combined with hand training consisting in 100% handgrip strength; (ii) hand training consisting in 100% and 50% of maximal handgrip strength combined with 90% eEmc intensity. The evaluations included box and blocks test (BBT), maximal voluntary contraction (MVC), F wave persistency, F/M ratio, spinal and cortical motor evoked potentials (MEP), recruitment curves of spinal MEP and cortical MEP and short-interval intracortical inhibition. The results showed that: (i) 90% eEmc intensity increased BBT, MVC, F wave persistency, F/M ratio and cortical MEP recruitment curve; 110% eEmc intensity increased BBT, F wave persistency and cortical MEP and recruitment curve of cortical MEP; (ii) 100% handgrip strength training significantly modulated MVC, F wave persistency, F/M wave and cortical MEP recruitment curve in comparison to 50% handgrip strength. In conclusion, eEmc intensity and muscle strength during training both influence the results for neuromodulation at the cervical level.

## 1. Introduction

Transcutaneous spinal cord stimulation (tSCS) used as a method of electrical enabling motor control (eEmc) is a novel, non-invasive method and alone or combined with hand training offers promise in improving hand function since it can modify the functional state of the sensory-motor system [1,2]. eEmc consists of low intensity electrical stimulation applied for changing the physiological states of spinal networks to a level that it enables the spinal network to better respond to voluntary commands and proprioceptive inputs. In the literature, the different terminologies have been used for transcutaneous spinal cord stimulation: “transcutaneous spinal stimulation (TSS) [3], painless transcutaneous electrical enabling motor control (pcEmc) [4], transcutaneous electrical stimulation of the spinal cord (TESS)” [5] or “transcutaneous enabling motor control (tEmc)” [6]. Recent developments in therapeutic approaches for spinal cord injury (SCI) showed that tSCS alone [3] or a combination of spinal cord stimulation with pharmacological treatment [7] or with hand training [6,8,9] allowed improving hand motor function in individuals with SCI. It has been hypothesized that spinal electrical stimulation can change the excitability of spinal circuitry and potentially neuromodulate the spinal network to facilitate and enhance the restoration of paralyzed limb function [5,6,7,8,9]. Plastic changes have been also reported in the control of upper limb function in healthy subjects following eEmc alone [5] and combined with hand training [10]. It has been suggested that the mechanisms recruited by eEmc combined with physical training, although partially overlapping, may involve different and perhaps synergistic processes leading to more effective reorganization of neural circuits [10]. It was recently reported that tSCS was capable of facilitating cortically evoked muscle responses and the degree of facilitation progressively increased during the 1 s stimulation training, and was still evident 0.5 s after the end of the training in monkeys [11]. The most likely mechanism of eEmc occurs via transcutaneous tonic spinal activation by elevating spinal networks excitability [12] and may affect interneuronal pathways that generate action potentials on motoneurons within a motor pool in a more normal stochastic time frame [13]. As such, eEmc is hypothesized to potentiate the generation of postsynaptic excitatory potentials and, thus, shift the spinal motor network excitability closer to the excitation threshold. In addition, activation of back musculature under the electrodes of tSCS [11] and of sensory afferents at the level of dorsal roots or via the spinal pathways can contribute to elevating neural excitability [14,15].

According to reports in the literature, there was great variability in the intensity of eEmc applied, though most of the studies used high intensities, close to the participants’ tolerance threshold. In cervical SCI, the stimulus intensity for eEmc varied from below the resting spinal motor threshold (RMT) to an intensity adjusted to enable maximal grip strength or adjusted based on the participant’s functional task performance [5,6,7,8,9]. When eEmc was combined with hand training, variable protocols were used: either maximal handgrip to submaximal isometric hand movement such as squeezing/grasping or standard stretching, active assistive range of motion exercises, intensive gross and fine motor skill training or the intensity that made the task easiest [6,8,9]. All these studies reported significant functional improvement of hand muscle strength and/or neurophysiological changes. We recently showed that even one single session of cervical eEmc is able to modify the excitability of neural networks controlling upper limb function in healthy subjects [10]. These changes strongly depend on the combination of eEmc with hand training, since improved hand grip force and increased spinal and corticospinal output were found in comparison to each intervention tested alone [10]. The intervention consisted in eEmc at an intensity of 90% the RMT at the cervical spinal cord and handgrip training at maximum voluntary contraction (MVC) [10]. However, it remains unknown if the intensity of electrical stimulation for eEmc and the level of hand grip strength in such combined strategy can affect motor strength and/or functional outcome, and if plastic changes occur at spinal or cortical level.

Thus, the objectives of this study were to test: (1) the effect of different electrical stimulation intensities for eEmc combined with the maximum force of hand grip during training, and (2) the effect of different hand grip strength during training combined with 90% of spinal RMT electrical stimulation for eEmc on hand function and spinal cord and cortical excitability. We hypothesized that eEmc at higher stimulus intensity applied at two sites of cervical spinal cord combined with a higher level of hand grip strength can enhance motor strength and functional outcome and eventually modulate spinal or cortical neural circuits more than lower stimulus intensity and lower hand grip strength during training.

## 2. Experimental Section

### 2.1. Study Design

Nine healthy volunteer subjects (age range 25–60 years; mean age 39.8 ± 11.1 years; 3 females and 6 males) participated in the study (Table 1). The inclusion criteria were: age between 18 and 65 years, without any neurological disorder and uncontrolled disease, which could limit the experiment, and given written informed consent. Exclusion criteria were any implanted metallic or electrical devices, and pregnancy. The study protocol was approved by the Research Ethics Committee of the Institute Guttmann and was conducted in accordance with the Declaration of Helsinki.

**Figure 1:**
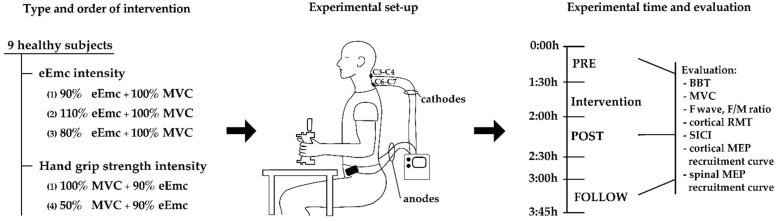
Schematic representation of the experiment conditions, order of intervention and the evaluation time points during each experiment, and the functional and motor strength assessment and neurophysiological assessments performed.

This study was performed in 2 parts. In the first part, we studied the effect of different electrical stimulation intensities for eEmc calculated from the spinal RMT of abductor pollicis brevis (APB) muscle combined with the maximum hand grip strength during training. In the second part, we studied the effect of different handgrip strength levels during training combined with 90% spinal RMT of stimulus intensity for eEmc (Figure 1).

### 2.2. Interventions

The study included a total of four interventions, tested in two parts separated at least one week apart. All interventions combined eEmc with hand training. In the first part, we studied the effect of three electrical eEmc stimulation at intensity 80%, 90% and 110% of spinal RMT of the APB muscle, combined with the maximum hand grip strength (100% of MVC). In the second part, we studied two different levels of hand grip strength for training, 50% and 100% of MVC, combined with eEmc at stimulus intensity of 90% of spinal RMT.

Each intervention consisted of trains of 20 s of concomitant eEmc stimulation and hand training, followed by 80 s of rest, for 30 min. The subject alternated the two hands during eEmc, resulting in nine hand-training attempts for each hand and 18 in total during the whole intervention [10].

The eEmc was delivered through two circular hydrogel adhesive electrodes (2 cm diameter, Axion GmbH, Leonberg, Germany) placed along the midline over spinous processes C3-C4 and C6-C7. eEmc was delivered using biphasic rectangular 1 ms pulses at a frequency of 30 Hz, with each pulse filled with a carrier frequency of 10 kHz [10]. For eEmc, we used two channels of a five-channel current-controlled stimulator of Biostim-5 stimulator (Cosyma Inc., Moscow, Russia) with two pairs of anode and cathode. Each channel was set up independently. Stimulation of the first channel was delivered by a cathode at C3-C4 level and stimulation of the second channel by a cathode at C6-C7, with anodes placed at the iliac crests.

The hand training consisted of holding the hand grip dynamometer and maintaining a hand grip strength of 100% of MVC for all stimulus intensities of eEmc in the first part. In the second part, either 100% (100% MVC) or 50% (50% MVC) of hand grip strength during hand training was combined with eEmc at 90% of stimulus intensity.

### 2.3. Functional and Motor Strength Assesment of the Healthy Participants

The evaluation protocol consisted of two hand functional outcomes, the Box and Block test (BBT) and the measurement of hand grip strength during a MVC with a dynamometer. Neurophysiological assessment was made on the dominant hand and arm muscles. The dominant hand was determined according to the Edinburgh inventory [16]. F-wave and spinal motor evoked potentials (spinal MEPs) in response to single-pulse C3-C4 and C6-C7 tSCS were recorded in the APB muscle to study the spinal cord excitability. At the cortical level, using transcranial magnetic stimulation (TMS), we determined the cortical RMT of APB muscle, and recorded cortical motor evoked potentials (cortical MEPs), short-interval intracortical inhibition (SICI) and recruitment curve of cortical MEPs. The neurophysiological studies were performed with an EMG machine (Medelec Synergy, Oxford Instruments; Surrey, England). The evaluation protocol was repeated at three time points: before (PRE), just after (POST) and one hour after finishing the intervention (FOLLOW) in each subject and experimental study (Figure 1). The total duration of each experiment was around four hours.

#### 2.3.1. Box and Block Test (BBT)

The BBT was used to assess hand dexterity. The participant was seated in front of a box split in two halves, the one on the preferred side full of small cube-shaped blocks [17]. The subject had to move the blocks one by one to the other side, crossing the middle line, during 60 s. The total number of moved blocks was the BBT score.

#### 2.3.2. Maximum Voluntary Contraction (MVC) during Hand Grip

The participant was seated in front of a table, with the dominant forearm resting on it in neutral position, holding a dynamometer (Jamar Model 5030J1, Sammons Preston, NJ, USA) with the hand. For trigger signal, we used a mild electric stimulus (3 mA intensity with pulse width 0.5 ms) delivered by means of ring electrodes around the fifth finger. Following the trigger signal, the participant had to perform a MVC and maintain it during 4 s; the strength was measured in kilograms (kg) in three consecutive trials, with at least one minute rest between them.

### 2.4. Neurophysiological Assesment

Disposable adhesive surface electrodes (outer diameter 20 mm; Technomed, Maastricht Airport, Netherlands) were placed over the muscle belly of the APB, abductor digiti minimi (ADM), flexor carpi radialis (FCR), extensor digitorum (ED) and biceps brachii (BB) muscles of the dominant arm, with the cathode proximal and the anode approximately 2 cm distally after standard skin preparation. The EMG signal was amplified, filtered using band-pass of 30 Hz–10 kHz, and epochs of 100 ms sweep duration at amplitude sensitivity of 0.1–0.5 mV recorded at a sampling rate of 50 kHz. EMG activity was visualized online, with the subject relaxing her/his arm and hand. If any background EMG activity was observed after stimulus delivery, the recording was discarded, and the stimulation was repeated. Offline analysis was performed with MATLAB.

#### 2.4.1. F Wave Persistency and F/M Wave Ratio

Suprathreshold electrical stimulation (pulse width 0.5 ms) of the median nerve at the wrist was used to elicit the maximum M wave and F wave in the APB muscle for at least 15 recordings. We assessed spinal motoneuron excitability using F-wave persistency and the Fmax/Mmax ratio (F/M ratio) in the APB muscle.

#### 2.4.2. Spinal MEP

Stimulation was delivered using two hydrogel adhesive disk electrodes (20 mm diameter; Axion GmbH, Germany) as cathodes along the midline between spinous processes C3-C4 and C6-C7. The anodes were two adhesive rectangular electrodes (5 × 12 cm) placed symmetrically over the iliac crest. Monophasic rectangular single pulses of 1 ms duration were delivered from a Biostim-5 stimulator (Cosyma Inc., Moscow, Russia). The spinal RMT was calculated as the lowest intensity of electrical stimulation applied first at C3-C4 and then at C6-C7 that evoked a spinal MEP of ≥50 μV peak-to-peak amplitude in the APB muscle in at least 5 of 10 consecutive trials. Then, the recruitment of spinal MEPs on all the muscles tested was recorded for each stimulation site and time point. Recruitment curves of spinal MEPs were derived from responses to gradually increasing stimulation intensity from 90% to 150% of the baseline spinal RMT at 10% increments, with three repetitions at each intensity.

#### 2.4.3. Transcranial Magnetic Stimulation (TMS)

A Magstim^®^ BiStim^2^ TMS (Magstim Company, Whitland, Wales, UK) apparatus was used. A figure-of-eight coil was held tangentially to the scalp over the motor area of the dominant hand in the optimal position for activating the APB in a posterior-anterior current direction. The hot point was defined as a point over the scalp where the largest amplitude cortical MEP in APB muscle was recorded. Subjects were seated in a chair, resting their pronated forearms on a desk in front of them and were asked to stay relaxed but awake throughout the test.

#### 2.4.4. Neurophysiological Parameters

We recorded the following parameters pre and post intervention (1) the BBT score to evaluate hand dexterity and (2) the MVC for handgrip strength. To evaluate spinal cord excitability changes: (1) F wave persistency and F/M wave ratio; (2) recruitment curve of spinal MEP. To evaluate cortical excitability changes: (1) cortical RMT, defined as the lowest intensity of TMS that evoked a cortical MEP of ≥50 μV peak-to-peak amplitude in the APB muscle in at least 5 of 10 consecutive trials; (2) mean amplitude of cortical MEPs using single-pulse TMS at 120% of cortical RMT of ABP in 5 recordings; (3) short intracortical inhibition (SICI) using paired-pulse TMS with a subthreshold conditioning stimulus (80% of cortical RMT) and a suprathreshold test stimulus (120% of cortical RMT) at interstimulus interval of 2 ms in 5 recordings without background activity [18]; (4) recruitment curves of cortical MEP obtained at increasing intensities from 90% to 150% of cortical RMT of APB, at 10% increments (three recordings at each intensity). The absence of baseline EMG activity was verified before carrying out each of the recordings; between 1 to 6 recordings for each subject were rejected because of background activity.

### 2.5. Data Analysis and Statistics

All data are expressed as the mean ± standard error of the mean (SEM), at baseline (PRE), just after (POST) and sixty minutes after finishing the intervention (FOLLOW) in each condition. We calculated the % changes with respect to baseline for each time point evaluation when appropriate. In the analysis of the recruitment curves, differences between pre vs. post intervention, and vs. follow were calculated. BBT was calculated just from one trial and MVC (hand muscle strength) was calculated as the mean of 3 trials.

For the F wave, the peak-to-peak amplitude and for Mmax, the maximum amplitude of the M wave was measured. F wave was considered if peak-to-peak amplitude was at least 1% of M wave (Mmax) amplitude. F wave persistence was calculated by dividing the number of F responses by the number of stimuli from 15 recordings. The maximum amplitude of F wave was normalized to Mmax amplitude to obtain Fmax/Mmax ratio.

For recruitment curves of spinal MEPs and cortical MEPs, we measured the peak-to-peak MEP amplitude in each recording, and then calculated the mean amplitude of MEPs from 3 recordings for each intensity and for each subject and for each experimental condition. Recruitment curves obtained at post and follow-up were normalized calculating the amplitude difference according to pre intervention recruitment curves values.

For SICI, averaged peak-to-peak amplitude of the conditioned MEP (obtained after the conditioning stimulus of 80% cortical RMT) was expressed as a percentage of the average amplitude of the test MEP (obtained at supramaximal 120% cortical RMT stimulus), according to % = (conditioned MEP/test MEP) * 100.

Shapiro–Wilk test was used to assess if data were normally distributed, and sphericity was evaluated with Mauchly’s test. Greenhouse–Geisser correction was used when the assumption of sphericity was violated (*p* < 0.05). All data was normally distributed, and a two-way repeated measures ANOVA (RM-ANOVA) was used for all statistical analyses to study the main effects of intervention and time on the functional and motor strength assessment and neurophysiological assessments for the raw and the normalized data. Interaction effect was also studied to determine if the interaction between intervention and time factors could affect the dependent variable. For significant interaction in the normalized data, time point and intervention simple effects were calculated and shown in results and figures. Time point multiple comparisons were performed with the Dunnett test (comparison between baseline vs. post and baseline vs. follow-up). The intervention multiple comparisons were compared with the Tukey test for the first part of the study (three interventions) and with Šidák test for the second part (two interventions). Significance level was set at *p* < 0.05 in all cases. Posterior estimation effect size η^2^ was calculated (0.01, 0.06, >0.14 as small, medium and large effect respectively).

## 3. Results

Demographic data of healthy individuals and used intensities for eEmc are given in Table 1. All volunteers finished the four experimental interventions with some discomfort sensation subjectively reported but not quantified, especially for the higher intensities during cervical stimulation, on the neck around the electrodes and slight extension of the neck because of cervical paravertebral muscle contraction.

### 3.1. Effects of eEmc Intensity

Raw data of functional and motor strength and neurophysiological assessment during the different electrical stimulus intensity for eEmc, in the first part of the study, are shown in Table 2.

#### 3.1.1. Functional and Motor Strength Assessment

**Figure 2:**
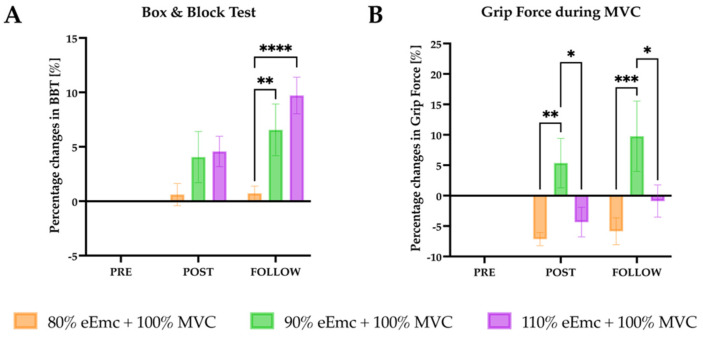
Functional and motor outcomes assessed by the Box and Block test and MVC. (**A**) percentage changes in the number of blocks moved in the Box and Block test with respect to baseline. Intervention simple effect shows significant differences at follow of 90% (^**^ *p* = 0.004) and 110% eEmc (^****^ *p* < 0.0001) with respect to 80% eEmc; (**B**) percentage changes in MVC with respect to baseline. Intervention simple effect showed differences of 90% eEmc compared with 80% (^**^ *p* = 0.0026) and 110% eEmc at post (^*^ *p* = 0.0211) and follow (^***^ *p* = 0.0108; ^*^ *p* = 0.0002) time points.

*Box and Block test*. There was a significant effect of time and interaction in the raw data (Table 2) and an effect of time, intervention and interaction in the normalized data. The percentage change of number of boxes during follow-up was significantly higher for 90% and 110% intensity of eEmc with respect to 80% (Figure 2A).

*Grip force during MVC*. RM-ANOVA showed significant effect of the time and interaction in the raw data; and effect of time, intervention and interaction in the normalized data. Intervention simple effect in percentage changes of MVC showed higher maximum muscle grip strength with 90% intensity of eEmc than with 110% and 80% at post intervention and follow-up time points (Figure 2B).

#### 3.1.2. Neurophysiological Assessment

**Figure 3:**
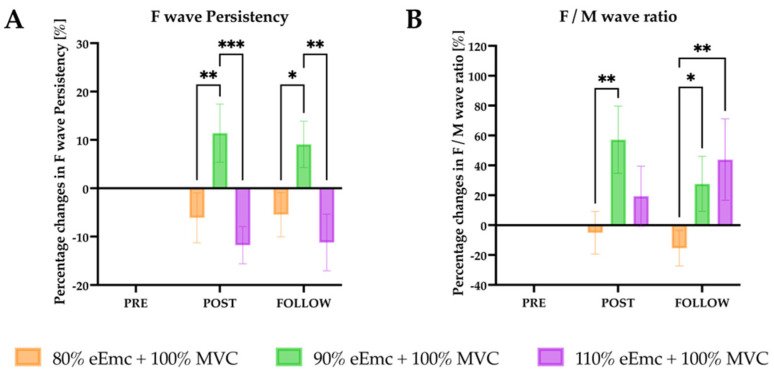
F wave persistency and F/M wave ratio. (**A**) percentage changes in F wave persistency with respect to baseline. Intervention simple effect showed significant differences of 90% eEmc with respect to 80% (^**^ *p* = 0.0079) and 110% eEmc (^***^ *p* = 0.0005) at post and at follow (^*^ *p* = 0.0304; ^**^ *p* = 0.0020); (**B**) percentage changes in F/M ratio with respect to baseline. Intervention simple effect showed significant differences at post of 90% and 80% eEmc (^**^ *p* = 0.0032); and at follow of 80% eEmc and 90% (^*^ *p* = 0.0491) and 110% eEmc (^**^ *p* = 0.0051).

*F-wave.* There was a significant effect of interaction on the persistence of the F wave in the raw data. The percentage change of F wave persistency was significantly higher for 90% of eEmc intensity at post intervention in comparison with 80% and 110%, and at follow-up in both 90% and 110% in comparison with 80% (Figure 3A).

For Mmax wave, RM-ANOVA showed significant effect of intervention in the raw data, but the percentage change of Mmax amplitude did not show any significant effect. On the other hand, the normalized Fmax/Mmax ratio showed significant effect of interaction. It was significantly higher with 90% of eEmc in comparison with 80% at post intervention; and with 90% and 110% in comparison with 80% of eEmc at follow-up evaluation (Figure 3B).

*Spinal MEPs recruitment curve.* RM-ANOVA showed no significant differences in the raw or the normalized data regarding results of the recruitment curve of spinal MEPs at C3-C4 or at C6-C7 level.

*Cortical RMT, TMS-induced cortical MEPs and SICI.* Cortical RMT and SICI did not change significantly at any eEmc stimulation intensity (*p* > 0.05) neither in the raw nor in the normalized data.

**Figure 4:**
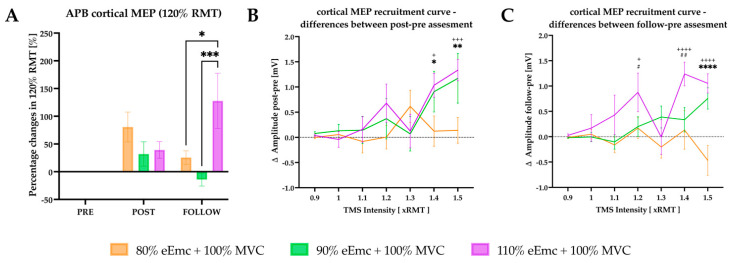
Cortical excitability outcomes. (**A**) percentage changes in cortical MEP evoked by TMS 120% of RMT with respect to baseline. Intervention simple effect showed significant differences at follow of 110% eEmc with respect to 80% (* *p* = 0.0126) and 90% eEmc (*** *p* = 0.0005); (**B**) difference in cortical MEP amplitude between post and pre time points. Intensity simple effects showed significant differences of 80% eEmc with respect 90% and 110% of eEmc at 1.4 (* *p* = 0.0390, ^+^ *p* = 0.0133) and 1.5 (** *p* = 0.0042, ^+++^ *p* = 0.0008) RMT cortical MEP; (**C**) difference in cortical MEP amplitude between follow and pre time points. Intensity simple effects showed significant differences of 80% and 110% eEmc at 1.2 (^+^ *p* = 0.0162), 1.4 (^++++^ *p* < 0.0001) and 1.5 RMT (^++++^ *p* < 0.0001); and of 90% and 110% eEmc at 1.2 (^#^ *p* < 0.0225) and 1.4 RMT multiple (^##^ *p* = 0.0015); and of 80% and 90% eEmc at 1.5 (**** *p* < 0.0001) RMT multiple.

For the TMS-induced cortical MEPs at 120% of RMT, RM-ANOVA showed significant effect of intervention and interaction in the raw data. However, there was significant effect of time, intervention and interaction in the normalized data. According to intervention simple effect, the percentage change of MEP amplitude was significantly higher at 110% of eEmc with respect to 80% and to 90% at follow-up (Figure 4A).

For recruitment curves of cortical MEP, there was a significant effect of intensity and interaction for difference between pre and post, and an effect of interaction for difference between pre and follow-up. We found a significantly higher MEP amplitude with 90% and 110% of eEmc compared with 80% at post intervention (Figure 4B). The MEP amplitude was significantly higher at follow-up testing in 90% and 110% of eEmc with respect to 80% for 1.4× and 1.5 × RMT (Figure 4C), and in 110% with respect to 80% and 90% of eEmc for 1.1 × RMT (Figure 4C).

### 3.2. Effects of Hand Grip Force during Training

Raw data of functional and motor strength assessment and neurophysiological assessments during the second part of the study, evaluating different hand grip strength levels for training, combined with a fixed eEMC intensity, are shown in Table 3.

#### 3.2.1. Functional and Motor Strength Assessment

*Box and Block test*. RM-ANOVA showed an effect of time and interaction in the raw data (Table 3), and only of time in the normalized data. The percentage of BBT changes was not significant between 50% vs. 100% handgrip strength condition.

**Figure 5:**
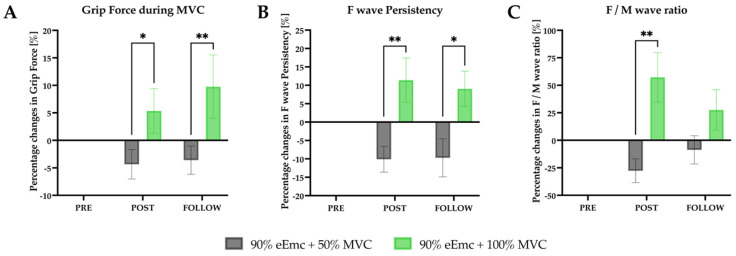
Changes in MVC, F wave persistency and F/M wave ratio. (**A**) percentage changes in grip force during MVC according to baseline. Intervention simple effect showed differences at post (* *p* = 0.0144) and follow (** *p* = 0.0011); (**B**) percentage changes in F wave persistency according to baseline. Intervention simple effects showed significant differences at post (** *p* = 0.0073) and follow (* *p* = 0.0191); (**C**) percentage changes in F/M ratio according to baseline. Intervention simple effect showed significant differences at post (** *p* = 0.0016).

*Grip force during MVC*. RM-ANOVA showed an effect of intervention and interaction in the raw and in the normalized data (Table 3). The percentage of MVC increased significantly in 100% handgrip strength condition compared with 50% at post intervention and at follow-up (Figure 5A).

#### 3.2.2. Neurophysiological Assessment

*F-wavepersistence*. RM-ANOVA showed effect of interaction in the raw data, and effect of intervention and interaction in the normalized data. According to intervention simple effects, the percentage of F wave persistency increased significantly with 100% hand grip strength compared to 50% at post intervention and follow-up (Figure 5B).

*F/M wave ratio*. RM-ANOVA showed no effect in the raw data but showed significant effect of intervention and interaction in the normalized data. There was a significantly higher ratio with 100% of handgrip strength intervention than with 50% at post intervention (Figure 5C).

*Spinal MEPs recruitment curve.* RM-ANOVA did not show any significant differences in raw and normalized data of the recruitment curve of spinal MEP evoked with stimulation either at C3-C4 or C6-C7 (*p* > 0.05).

**Figure 6:**
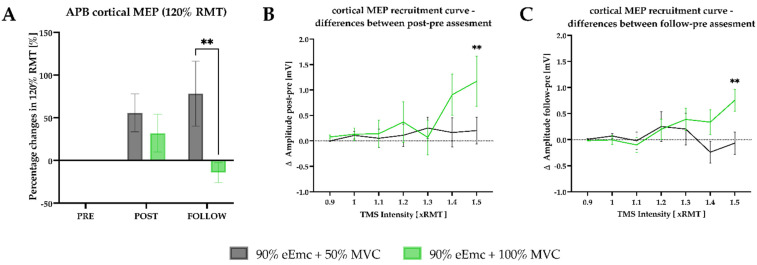
Cortical excitability outcomes. (**A**) percentage changes in cortical MEP from 120% of RMT according to baseline. Intervention simple effect showed significant differences at follow; (**B**) difference amplitude between post and pre cortical MEP recruitment. Intensity simple effects showed significant differences at 1.5 RMT multiple (** *p* = 0.0059); (**C**) difference amplitude between follow and pre MEP recruitment. Intensity simple effects showed significant differences at 1.5 RMT multiple (** *p* = 0.0063).

*Cortical RMT, TMS-induced cortical MEPs and SICI.* RM-ANOVA showed significant effect of time in the raw and in the normalized data for cortical RMT, but not for SICI. On the other hand, TMS-induced cortical MEPs at 120% RMT showed significant effect of interaction in the raw and in the normalized data. The cortical MEP amplitude was higher in 50% than in 100% handgrip strength intervention at follow-up (Figure 6A).

In the recruitment curve of cortical MEPs, RM-ANOVA showed significant effect of intensity and interaction between pre and post, and effect of interaction between pre and follow-up. Additionally, 100% of handgrip strength intervention significantly increased the cortical MEP amplitude at 1.5 × RMT with respect to 50% of MVC at post intervention and at follow-up (Figure 6B,C).

## 4. Discussion

In a previous study we reported that eEmc combined with hand training improves hand grip force and increases spinal and corticospinal excitability in comparison to each intervention tested alone [8]. In the present study, we further investigated if the levels of both eEmc intensity and muscle strength during training could influence the results for neuromodulation at the cervical spinal cord. The results of this study show that eEmc at 90% intensity of spinal RMT of the APB muscle combined with maximal handgrip strength induced better hand function and muscle strength than the other conditions assayed. The results of F wave persistency and F/M ratio and of cortical MEP recruitment curves reflected stronger plastic changes in the spinal and cortical pathways of eEmc at 90% intensity compared to eEmc at intensities of 80% or 110%. Most of these effects were maintained at least for one hour following the intervention. On the other hand, eEmc at 110% intensity induced better hand function and higher F/M ratio and cortical plasticity changes with respect to eEmc at 80%. In contrast, eEmc at 80% of intensity did not show any noticeable effect on hand function, spinal and cortical excitability. In the second part of the study, eEmc at 90% intensity was combined with training at the maximal (100%) or at half (50%) MVC in hand grip strength. With 100% MVC, there was higher muscle strength and more plastic changes at spinal cord, measured by F wave persistency and F/M ratio, and at cortical level, measured by cortical MEP recruitment curve, than with 50% MVC. Most of these effects were also maintained at least one hour during follow-up testing. In contrast, with the 50% MVC of grip strength there was only a significant increase in cortical excitability measured by cortical MEP.

### 4.1. Effects of eEmc Intensity

There is no consensus in the literature about which intensity of electrical stimulation may modulate more effectively the functional outputs of the cervical spinal cord. Thus, previous studies have used different methods to select the eEmc intensity. In our previous study in healthy individuals, we empirically used a stimulation intensity at 90% of spinal RMT of the APB muscle [10], whereas Benavides et al. [5] had used 100% of RMT of the Biceps brachii muscle in SCI and healthy individuals. Gad et al. [6] adjusted the eEmc to enable maximal grip strength or submaximal isometric hand movement without causing discomfort in SCI subjects. Freverty et al. [7] used varying combinations of eEmc parameters to obtain optimal facilitation of voluntary hand grip by identification of the relative activation levels of the motor pools, and combined eEmc with medication (Buspirone) without hand training. Studying cervical SCI subjects, other authors used different strategies, such as beginning eEmc at 50 mA and adjusting the intensity based on the functional task performance and subjective feedback during the intervention [9], increasing stimulus intensity in 10 mA intervals from 10 to 120 mA [8], or from 5 to 68 mA [19].

Despite the different stimulus intensity used for eEmc, the most likely direct mechanism of eEmc occurs via transcutaneous tonic spinal activation by elevating spinal networks excitability [12] and may activate interneuronal pathways that generate action potentials on motoneurons within a motor pool in a more normal stochastic time frame [13]. As such, eEmc is hypothesized to potentiate the generation of postsynaptic excitatory potentials and thus shift the spinal motor network excitability closer to the excitation threshold. The constantly changing postsynaptic potentials intrinsic to spinal networks [20] can contribute to a constantly changing population of interneurons being asynchronously activated in a random pattern [21]. According to our results, it can be interpreted that eEmc, at higher levels when delivered at 90% stimulus intensity, brings interneurons and motoneurons closer to motor threshold and, therefore, more likely to respond to descending drive, and elevates the excitability of spinal cord networks and possibly also of motor cortex. In addition, sensory afferents at the level of dorsal roots and/or the dorsal column spinal pathways may be affected by the tSCS and thus contribute differentially to elevating or suppressing spinal and/or supraspinal neural excitability [14,15,21]. Despite we cannot preclude this mechanism, considering that the stimulation was applied at C3 level, far cranial from the APB muscle root innervation segment (C8-T1), we hypothesize that the effect on muscle response is more likely due to stimulation of descending pathways and premotoneuronal network than to the stimulation of proprioceptive fibers within posterior roots.

From our results, it appears that there is a window of intensity for the neuromodulatory effects of eEmc. The effects of an eEmc intensity of 90% of spinal RMT of the target muscle exceeded the effects of lower (80%) and higher (110%) stimulus intensities. It is unclear, however, why only the 90% level of intensity enabled higher voluntarily controlled drive to produce stronger contractions of hand training, which subsequently strengthens the neuromuscular network. It is, noteworthy that eEmc at 110% intensity augmented hand muscle strength less than 90% intensity but induced more plastic changes at cortical level than 90% and 80% intensities. Perhaps the higher eEmc intensity can elicit impulses that are conducted antidromically to the cortex or induce a greater facilitatory effect among brain networks, inducing more effectively plastic changes at the cortical than the spinal level. Thus, our data suggest that eEmc at suprathreshold intensity modulates more effectively the cortical excitability than at subthreshold intensities, but hand function, muscle strength and spinal cord excitability were modulated more effectively by subthreshold intensity of 90% spinal RMT.

### 4.2. Effects of Handgrip Strength during Training

Our results indicate that the combination of maximal (100% of MVC) handgrip strength with eEmc further increases the hand muscle strength and induces more effectively plastic changes at spinal cord and cortical levels in comparison to lower (50%) handgrip strength.

There is no consensus either about what kind of hand training could help to modulate, more effectively, spinal cord motor function in healthy individuals or following SCI. Indeed, only a few studies on eEmc at cervical cord level combined tSCS with hand training. In the study by Gad et al. [6] with cervical SCI subjects, two tasks were performed: isometric maximum handgrip contraction or voluntary rhythmic efforts of submaximal contraction (opening and closing the hand). In another study on a single SCI subject, a functional task combined with eEmc aimed to address hand grasp and pinch bilaterally [9]. In healthy individuals, we previously used maximal handgrip force for hand training combined with eEmc [10]. Several observations made after a single training or stimulation session suggest that short-term plastic changes occur [5,10,22]. Performing the maximum handgrip strength combined with eEmc at 90% of stimulus intensity modulates more effectively hand motor function and induces more effectively neurophysiological changes at spinal cord and cortical levels than when performing lower hand grip strength.

According to our results, activity-based plasticity depends on the level of hand grip strength for hand training and the eEmc intensity for neuromodulation. Activity-based neural plasticity mechanisms involve both functional modifications of existing synapses and structural changes that alter the synaptic connectivity of neurons (formation, removal and remodeling of synaptic buttons and even dendrites) [22]. A single exposure to activity-dependent plasticity by physical training combined with electrical stimulation may induce these short-term changes that in turn could promote occurrence of long-term plasticity under repeated exposure that involves mechanisms such as long-term potentiation and depression, morphological changes of dendrites, synaptogenesis and axonal branching/regeneration [23].

### 4.3. Limitations of the Study

It has to be noted that the sample size in this study was relatively small, limiting statistical power and interpretation of the results. Four days of experiments and the long duration of each experiment may result in fatigue factors, limiting the number of replicates that could be recorded for neurophysiological parameters, resulting in more variability. The only follow-up assessment period was for one hour after the end of session, thus providing no comparison for other longer timeframes. Nevertheless, the obtained results open a work frame for further studies refining the conditions of combinatory neuromodulation for SCI patients.

## 5. Conclusions

Taken together, this study demonstrates an approach to assess two modulatory interventions, eEmc and activity-dependent intervention at the supraspinal, and spinal and peripheral levels. It also provides basic physiological responses in healthy subjects, which will help the design of future studies involving other dysfunctional conditions. The findings of the study indicate that the stimulus intensity for eEmc is relevant for improving hand function and/or muscle strength, as well as hand muscle strength level and recruitment of motor units within a motor pool during training in conjunction with electrical neuromodulation. The data also demonstrate both the immediacy and short-term persistence of at least one hour after intervention. Interestingly, our results also allow us to hypothesize that spinal and cortical excitability may be modulated differently depending on the eEmc intensity. Therefore, both the electrical stimulus intensity of eEmc and the intensity of the voluntary effort during training should be considered when optimizing rehabilitation protocols for restoring motor performance.

## Figures and Tables

**Table 1:** Demographic data of healthy individuals and current intensities used for cervical stimulation at C3-C4 and at C6-C7 level.

				eEmc Intensity Applied during Stimulation (mA)							
				C3-C4				C6-C7			
				% eEmc + 100% MVC			90%eEmc + 50% MVC	%eEmc + 100%MVC			90%eEmc + 50% MVC
Subject	Sex	Age	Hand	80%	90%	110%		80%	90%	110%	
1	M	44	R	32	29	40	29	34	38	44	38
2	M	60	R	69	59	77	63	72	72	90	72
3	F	25	R	27	30	33	29	32	30	40	32
4	F	27	L	32	34	44	34	36	36	46	41
5	F	33	R	37	54	53	47	48	63	62	58
6	M	41	R	27	36	37	31	38	45	42	34
7	M	51	R	51	52	75	63	61	63	90	81
8	M	39	R	48	59	77	49	56	72	88	59
9	M	38	B	45	67	73	61	53	77	75	85

M: male; F: female; L: left; R: right; B: both.

**Table 2:** Raw data and statistics of functional and motor strength and neurophysiological assessment during the electrical stimulus intensity for eEmc at 80%, 90% and 110% combined with maximum (100%) handgrip strength during hand training.

	Time	80%	90%	110%	F (DFn, DFd)	*p* Value	η^2^
BBT (blocks number)	Pre	74.1 ± 3.0	72.8 ± 3.1	71.2 ± 2.7	Ftime (2, 16) = 17.32	<0.0001	0.033
	Post	74.6 ± 3.1	75.7 ± 3.5	74.4 ± 2.9	Finterven. (2, 16) = 0.16	0.855	0.002
	Foll.	74.6 ± 3.2	77.4 ± 3.4	78.0 ± 2.7	Finterac. (4, 32) = 3.49	0.018	0.014
MVC Grip Force (kg)	Pre	37.8 ± 8.3	31.3 ± 11.3	36.9 ± 7.6	Ftime (2, 16) = 5.87	0.012	0.002
	Post	35.1 ± 7.6	32.7 ± 11.0	35.5 ± 8.7	Finterven. (2, 16) = 3.59	0.052	0.040
	Foll.	35.8 ± 9.2	33.6 ± 9.8	36.4 ± 7.5	Finterac. (4, 32) = 2.71	0.048	0.009
F wave Persistency	Pre	65.4 ± 7.9	48.8 ± 9.1	71.5 ± 6.5	Ftime (2, 16) = 0.33	0.725	0.002
	Post	59.3 ± 8.5	60.2 ± 8.2	59.7 ± 8.0	Finterven. (2, 16) = 3.33	0.062	0.019
	Foll.	59.9 ± 9.5	57.9 ± 9.2	60.2 ± 10.1	Finterac. (4, 32) = 2.92	0.031	0.031
Mmax wave (microV)	Pre	13,296.3 ± 1754.9	16,829.0 ± 1863.2	16,844.5 ± 1204.9	Ftime (2, 16) = 1.77	0.202	0.018
	Post	12,752.8 ± 1427.5	15,485.3 ± 1937.6	16,234.6 ± 1579.3	Finterven. (2, 16) = 4.85	0.023	0.119
	Foll.	13,697.6 ± 1726.9	17,153.6 ± 1889.8	18,452.1 ± 970.3	Finterac. (4, 32) = 0.29	0.882	0.003
Ratio Fmax/Mmax	Pre	5.5 ± 0.8	3.7 ± 0.8	4.2 ± 0.6	Ftime (1.92, 15.33) = 0.15	0.851	0.001
	Post	4.6 ± 0.7	5.0 ± 0.8	4.5 ± 0.5	Finterven. (1.38, 11.03) = 0.55	0.530	0.008
	Foll.	4.2 ± 0.6	4.3 ± 0.7	5.4 ± 0.6	Finterac. (1.83, 14.64) = 1.93	0.082	0.069
RMT TMS	Pre	38.7 ± 2.3	39.1 ± 2.6	39.1 ± 2.0	Ftime (1.85, 14.81) = 1.46	0.263	0.002
	Post	37.8 ± 2.4	37.8 ± 2.4	39.3 ± 2.5	Finterven. (1.89, 15.14) = 0.21	0.805	0.002
	Foll.	38.2 ± 2.4	39.1 ± 2.6	38.4 ± 2.0	Finterac. (1.91,15.31) = 1.46	0.263	0.003
SICI in APB (%)	Pre	13.1 ± 3.7	13.8 ± 2.9	8.3 ± 1.2	Ftime (1.13, 9.05) = 0.93	0.374	0.021
	Post	10.8 ± 2.6	12.4 ± 4.2	7.1 ± 2.6	Finterven. (1.51, 12.07) = 0.14	0.816	0.003
	Follow	13.2 ± 4.2	16.7 ± 4.7	11.2 ± 4.1	Finterac. (1.90, 15.21) = 0.34	0.708	0.010
Cortical MEP at 120% RMT in APB (mV)	Pre	0.6 ± 0.1	0.8 ± 0.1	0.8 ± 0.1	Ftime (2, 16) = 3.43	0.058	0.091
	Post	1.1 ± 0.2	0.9 ± 0.1	1.1 ± 0.2	Finterven. (2, 16) = 5.25	0.018	0.056
	Foll.	0.8 ± 0.1	0.7 ± 0.1	1.6 ± 0.3	Finterac. (4, 32) = 4.99	0.003	0.113
**Stimulus Intensity**		**80%**	**90%**	**110%**			
TMS recruitment: diff post-pre (mV)	0.9	−0.002 ± 0.08	0.08 ± 0.11	0.04 ± 0.08	F_intensity (6, 48)_ = 10.69 F_interven. (2, 16)_ = 0.74 F_interac. (12, 96)_ = 2.17	<0.001 0.491 0.019	0.121 0.030 0.079
	1	0.06 ± 0.20	0.13 ± 0.37	−0.04 ± 0.47			
	1.1	−0.08 ± 0.68	0.14 ± 0.80	0.16 ± 0.77			
	1.2	0.004 ± 0.71	0.37 ± 1.20	0.68 ± 1.14			
	1.3	0.62 ± 0.96	0.07 ± 1.02	0.12 ± 1.00			
	1.4	0.124 ± 0.90	0.91 ± 1.21	1.03 ± 0.71			
	1.5	0.14 ± 0.77	1.17 ± 1.48	1.34 ± 0.64			
TMS recruitment: diff foll-pre (mV)	0.9	−0.01 ± 0.06	−0.02 ± 0.03	0.03 ± 0.09	F_intensity (6, 48)_ = 4.09 F_interven. (2, 16)_ = 3.61 F_interac. (12, 96)_ = 3.73	0.002 0.051 <0.001	0.078 0.102 0.109
	1	0.05 ± 0.28	−0.01 ± 0.25	0.17 ± 0.80			
	1.1	−0.16 ± 0.46	−0.10 ± 0.43	0.43 ± 1.18			
	1.2	0.17 ± 0.61	0.20 ± 0.59	0.88 ± 1.15			
	1.3	−0.20 ± 0.66	0.39 ± 0.65	−0.01 ± 1.03			
	1.4	0.13 ± 1.14	0.34 ± 0.72	1.24 ± 0.70			
	1.5	−0.47 ± 0.88	0.76 ± 0.63	1.05 ± 0.57			
C3-C4 recruitment: diff post-pre (mV)	0.9	0.02 ± 0.07	−0.004 ± 0.01	0.01 ± 0.02	F_intensity (6, 48)_ = 0.96 F_interven. (2, 16)_ = 0.99 F_interac. (12, 96)_ = 1.69	0.460 0.395 0.081	0.023 0.057 0.037
	1	0.01 ± 0.09	−0.01 ± 0.02	0.02 ± 0.05			
	1.1	0.01 ± 0.04	−0.03 ± 0.11	0.001 ± 0.03			
	1.2	0.01 ± 0.03	−0.04 ± 0.11	0.004 ± 0.02			
	1.3	−0.03 ± 0.09	−0.04 ± 0.11	−0.01 ± 0.03			
	1.4	0.03 ± 0.09	−0.06 ± 0.11	0.01 ± 0.04			
	1.5	0.04 ± 0.10	−0.003 ± 0.09	−0.02 ± 0.04			
C3-C4 recruitment: diff foll-pre (mV)	0.9	0.03 ± 0.09	0.02 ± 0.05	0.01 ± 0.02	F_intensity (6, 48)_ = 0.31 F_interven. (2, 16)_ = 0.78 F_interac. (12, 96)_ = 1.10	0.929 0.474 0.373	0.008 0.036 0.035
	1	0.02 ± 0.09	−0.01 ± 0.03	0.04 ± 0.10			
	1.1	0.01 ± 0.05	−0.002 ± 0.11	0.01 ± 0.02			
	1.2	0.02 ± 0.05	0.004 ± 0.02	0.02 ± 0.03			
	1.3	0.03 ± 0.04	0.01 ± 0.02	0.001 ± 0.03			
	1.4	0.04 ± 0.08	−0.01 ± 0.05	0.01 ± 0.03			
	1.5	0.06 ± 0.08	0.001 ± 0.04	0.01 ± 0.03			
C6-C7 recruitment: diff post-pre (mV)	0.9	0.003 ± 0.03	−0.001 ± 0.03	0.01 ± 0.02	F_intensity (6, 48)_ = 2.14 F_interven. (2, 16)_ = 0.67 F_interac. (12, 96)_ = 0.51	0.066 0.525 0.906	0.041 0.023 0.023
	1	−0.001 ± 0.03	0.04 ± 0.10	0.01 ± 0.03			
	1.1	0.004 ± 0.02	−0.01 ± 0.05	0.01 ± 0.03			
	1.2	−0.03 ± 0.09	−0.01 ± 0.06	−0.01 ± 0.03			
	1.3	−0.04 ± 0.11	−0.02 ± 0.04	0.001 ± 0.05			
	1.4	−0.03 ± 0.08	−0.01 ± 0.05	−0.004 ± 0.05			
	1.5	−0.02 ± 0.12	0.01 ± 0.04	0.01 ± 0.06			
C6-C7 recruitment: diff foll-pre (mV)	0.9	0.01 ± 0.02	−0.004 ± 0.03	0.02 ± 0.02	F_intensity (6, 48)_ = 0.40 F_interven. (2, 16)_ = 0.16 F_interac. (12, 96)_ = 0.72	0.878 0.850 0.725	0.010 0.005 0.039
	1	−0.0004 ± 0.03	0.03 ± 0.09	0.02 ± 0.02			
	1.1	0.01 ± 0.02	−0.001 ± 0.04	0.02 ± 0.04			
	1.2	−0.02 ± 0.10	0.01 ± 0.02	0.003 ± 0.03			
	1.3	0.02 ± 0.04	0.01 ± 0.02	−0.02 ± 0.08			
	1.4	0.01 ± 0.09	0.004 ± 0.03	0.004 ± 0.04			
	1.5	−0.01 ± 0.14	0.002 ± 0.02	0.03 ± 0.06			

diff post-pre: differences between post-pre evaluation; diff foll-pre: differences between baseline-follow up evaluation.

**Table 3:** Raw data and statistics of functional and motor strength and neurophysiological assessment during 100% or 50% of hand grip strength during hand training combined with the 90% of electrical stimulus intensity for eEmc.

	Time	80%	90%	110%	F (DFn, DFd)	*p* Value	η^2^
BBT (blocks number)	Pre	74.1 ± 3.0	72.8 ± 3.1	71.2 ± 2.7	Ftime (2, 16) = 17.32	**<0.0001**	0.033
	Post	74.6 ± 3.1	75.7 ± 3.5	74.4 ± 2.9	Finterven. (2, 16) = 0.16	**0.855**	0.002
	Foll.	74.6 ± 3.2	77.4 ± 3.4	78.0 ± 2.7	Finterac. (4, 32) = 3.49	**0.018**	0.014
MVC Grip Force (kg)	Pre	37.8 ± 8.3	31.3 ± 11.3	36.9 ± 7.6	Ftime (2, 16) = 5.87	**0.012**	0.002
	Post	35.1 ± 7.6	32.7 ± 11.0	35.5 ± 8.7	Finterven. (2, 16) = 3.59	**0.052**	0.040
	Foll.	35.8 ± 9.2	33.6 ± 9.8	36.4 ± 7.5	Finterac. (4, 32) = 2.71	**0.048**	0.009
F wave Persistency	Pre	65.4 ± 7.9	48.8 ± 9.1	71.5 ± 6.5	Ftime (2, 16) = 0.33	**0.725**	0.002
	Post	59.3 ± 8.5	60.2 ± 8.2	59.7 ± 8.0	Finterven. (2, 16) = 3.33	0.062	0.019
	Foll.	59.9 ± 9.5	57.9 ± 9.2	60.2 ± 10.1	Finterac. (4, 32) = 2.92	0.031	0.031
Mmax wave (microV)	Pre	13,296.3 ± 1754.9	16,829.0 ± 1863.2	16,844.5 ± 1204.9	Ftime (2, 16) = 1.77	0.202	0.018
	Post	12,752.8 ± 1427.5	15,485.3 ± 1937.6	16,234.6 ± 1579.3	Finterven. (2, 16) = 4.85	0.023	0.119
	Foll.	13,697.6 ± 1726.9	17,153.6 ± 1889.8	18,452.1 ± 970.3	Finterac. (4, 32) = 0.29	0.882	0.003
Ratio Fmax/Mmax	Pre	5.5 ± 0.8	3.7 ± 0.8	4.2 ± 0.6	Ftime (1.92, 15.33) = 0.15	0.851	0.001
	Post	4.6 ± 0.7	5.0 ± 0.8	4.5 ± 0.5	Finterven. (1.38, 11.03) = 0.55	0.530	0.008
	Foll.	4.2 ± 0.6	4.3 ± 0.7	5.4 ± 0.6	Finterac. (1.83, 14.64) = 1.93	0.082	0.069
RMT TMS	Pre	38.7 ± 2.3	39.1 ± 2.6	39.1 ± 2.0	Ftime (1.85, 14.81) = 1.46	0.263	0.002
	Post	37.8 ± 2.4	37.8 ± 2.4	39.3 ± 2.5	Finterven. (1.89, 15.14) = 0.21	0.805	0.002
	Foll.	38.2 ± 2.4	39.1 ± 2.6	38.4 ± 2.0	Finterac. (1.91, 15.31) = 1.46	0.263	0.003
SICI in APB (%)	Pre	13.1 ± 3.7	13.8 ± 2.9	8.3 ± 1.2	Ftime (1.13, 9.05) = 0.93	0.374	0.021
	Post	10.8 ± 2.6	12.4 ± 4.2	7.1 ± 2.6	Finterven. (1.51, 12.07) = 0.14	0.816	0.003
	Follow	13.2 ± 4.2	16.7 ± 4.7	11.2 ± 4.1	Finterac. (1.90, 15.21) = 0.34	0.708	0.010
Cortical MEP at 120% RMT in APB (mV)	Pre	0.6 ± 0.1	0.8 ± 0.1	0.8 ± 0.1	Ftime (2, 16) = 3.43	0.058	0.091
	Post	1.1 ± 0.2	0.9 ± 0.1	1.1 ± 0.2	Finterven. (2, 16) = 5.25	0.018	0.056
	Foll.	0.8 ± 0.1	0.7 ± 0.1	1.6 ± 0.3	Finterac. (4, 32) = 4.99	0.003	0.113
**Stimulus Intensity**		**80%**	**90%**	**110%**			
TMS recruitment: diff post-pre (mV)	0.9	−0.002 ± 0.08	0.08 ± 0.11	0.04 ± 0.08	F_intensity (6, 48)_ = 10.69 F_interven. (2, 16)_ = 0.74 F_interac. (12, 96)_ = 2.17	**<0.001** 0.491 **0.019**	0.121 0.030 0.079
	1	0.06 ± 0.20	0.13 ± 0.37	−0.04 ± 0.47			
	1.1	−0.08 ± 0.68	0.14 ± 0.80	0.16 ± 0.77			
	1.2	0.004 ± 0.71	0.37 ± 1.20	0.68 ± 1.14			
	1.3	0.62 ± 0.96	0.07 ± 1.02	0.12 ± 1.00			
	1.4	0.124 ± 0.90	0.91 ± 1.21	1.03 ± 0.71			
	1.5	0.14 ± 0.77	1.17 ± 1.48	1.34 ± 0.64			
TMS recruitment: diff foll-pre (mV)	0.9	−0.01 ± 0.06	−0.02 ± 0.03	0.03 ± 0.09	F_intensity (6, 48)_ = 4.09 F_interven. (2, 16)_ = 3.61 F_interac. (12, 96)_ = 3.73	**0.002** 0.051 **<0.001**	0.078 0.102 0.109
	1	0.05 ± 0.28	−0.01 ± 0.25	0.17 ± 0.80			
	1.1	−0.16 ± 0.46	−0.10 ± 0.43	0.43 ± 1.18			
	1.2	0.17 ± 0.61	0.20 ± 0.59	0.88 ± 1.15			
	1.3	−0.20 ± 0.66	0.39 ± 0.65	−0.01 ± 1.03			
	1.4	0.13 ± 1.14	0.34 ± 0.72	1.24 ± 0.70			
	1.5	−0.47 ± 0.88	0.76 ± 0.63	1.05 ± 0.57			
C3-C4 recruitment: diff post-pre (mV)	0.9	0.02 ± 0.07	−0.004 ± 0.01	0.01 ± 0.02	F_intensity (6, 48)_ = 0.96 F_interven. (2, 16)_ = 0.99 F_interac. (12, 96)_ = 1.69	0.460 0.395 0.081	0.023 0.057 0.037
	1	0.01 ± 0.09	−0.01 ± 0.02	0.02 ± 0.05			
	1.1	0.01 ± 0.04	−0.03 ± 0.11	0.001 ± 0.03			
	1.2	0.01 ± 0.03	−0.04 ± 0.11	0.004 ± 0.02			
	1.3	−0.03 ± 0.09	−0.04 ± 0.11	−0.01 ± 0.03			
	1.4	0.03 ± 0.09	−0.06 ± 0.11	0.01 ± 0.04			
	1.5	0.04 ± 0.10	−0.003 ± 0.09	−0.02 ± 0.04			
C3-C4 recruitment: diff foll-pre (mV)	0.9	0.03 ± 0.09	0.02 ± 0.05	0.01 ± 0.02	F_intensity (6, 48)_ = 0.31 F_interven. (2, 16)_ = 0.78 F_interac. (12, 96)_ = 1.10	0.929 0.474 0.373	0.008 0.036 0.035
	1	0.02 ± 0.09	−0.01 ± 0.03	0.04 ± 0.10			
	1.1	0.01 ± 0.05	−0.002 ± 0.11	0.01 ± 0.02			
	1.2	0.02 ± 0.05	0.004 ± 0.02	0.02 ± 0.03			
	1.3	0.03 ± 0.04	0.01 ± 0.02	0.001 ± 0.03			
	1.4	0.04 ± 0.08	−0.01 ± 0.05	0.01 ± 0.03			
	1.5	0.06 ± 0.08	0.001 ± 0.04	0.01 ± 0.03			
C6-C7 recruitment: diff post-pre (mV)	0.9	0.003 ± 0.03	−0.001 ± 0.03	0.01 ± 0.02	F_intensity (6, 48)_ = 2.14 F_interven. (2, 16)_ = 0.67 F_interac. (12, 96)_ = 0.51	0.066 0.525 0.906	0.041 0.023 0.023
	1	−0.001 ± 0.03	0.04 ± 0.10	0.01 ± 0.03			
	1.1	0.004 ± 0.02	−0.01 ± 0.05	0.01 ± 0.03			
	1.2	−0.03 ± 0.09	−0.01 ± 0.06	−0.01 ± 0.03			
	1.3	−0.04 ± 0.11	−0.02 ± 0.04	0.001 ± 0.05			
	1.4	−0.03 ± 0.08	−0.01 ± 0.05	−0.004 ± 0.05			
	1.5	−0.02 ± 0.12	0.01 ± 0.04	0.01 ± 0.06			
C6-C7 recruitment: diff foll-pre (mV)	0.9	0.01 ± 0.02	−0.004 ± 0.03	0.02 ± 0.02	F_intensity (6, 48)_ = 0.40 F_interven. (2, 16)_ = 0.16 F_interac. (12, 96)_ = 0.72	0.878 0.850 0.725	0.010 0.005 0.039
	1	−0.0004 ± 0.03	0.03 ± 0.09	0.02 ± 0.02			
	1.1	0.01 ± 0.02	−0.001 ± 0.04	0.02 ± 0.04			
	1.2	−0.02 ± 0.10	0.01 ± 0.02	0.003 ± 0.03			
	1.3	0.02 ± 0.04	0.01 ± 0.02	−0.02 ± 0.08			
	1.4	0.01 ± 0.09	0.004 ± 0.03	0.004 ± 0.04			
	1.5	−0.01 ± 0.14	0.002 ± 0.02	0.03 ± 0.06			

diff post-pre: differences between post-pre evaluation; diff foll-pre: differences between baseline-follow up evaluation.

## Data Availability

The data presented in this study are available on request from the corresponding author. The data are not publicly available due to privacy restrictions.
